# Supplementary material for: Exclusion of enrolled participants in randomised controlled trials: what to do with ineligible participants?
Source: BMJ Open. 2020 Dec 2;10(12):e039546. doi: 10.1136/bmjopen-2020-039546 (PMC7713189; doi:10.1136/bmjopen-2020-039546)
Supplement: Author's manuscript [file bmjopen-2020-039546.draft_revisions.pdf]

BMJ Open is committed to open peer review. As part of this commitment we make the peer review history of every article we publish publicly available.

When an article is published we post the peer reviewers' comments and the authors' responses online. We also post the versions of the paper that were used during peer review. These are the versions that the peer review comments apply to.

The versions of the paper that follow are the versions that were submitted during the peer review process. They are not the versions of record or the final published versions. They should not be cited or distributed as the published version of this manuscript.

BMJ Open is an open access journal and the full, final, typeset and author-corrected version of record of the manuscript is available on our site with no access controls, subscription charges or pay-per-view fees (http://bmjopen.bmj.com).

If you have any questions on BMJ Open's open peer review process please email info.bmjopen@bmj.com

BMJ Open

Exclusion of enrolled participants in randomised controlled trials: what to do with ineligible participants?

Journal:	*BMJ Open*
Manuscript ID	bmjopen-2020-039546.R1
Article Type:	Communication
Date Submitted by the Author:	11-May-2020
Complete List of Authors:	Rehman, Andrea; London School of Hygiene and Tropical Medicine, MRC Tropical Epidemiology Group<br>Ferrand, Rashida; London School of Hygiene & Tropical Medicine, Department of Clinical Research; Biomedical Research and Training Institute<br>Allen, Elizabeth; London School of Hygiene & Tropical Medicine, Department of Medical Statistics<br>Simms, Victoria; London School of Hygiene and Tropical Medicine, MRC Tropical Epidemiology Group; Biomedical Research and Training Institute<br>McHugh, Grace; Biomedical Research and Training Institute, Harare<br>Weiss, Helen; London School of Hygiene and Tropical Medicine, MRC Tropical Epidemiology Group
**Primary Subject Heading**:	Research methods
Secondary Subject Heading:	Epidemiology
Keywords:	STATISTICS & RESEARCH METHODS, Paediatric thoracic medicine < THORACIC MEDICINE, EDUCATION & TRAINING (see Medical Education & Training)

SCHOLARONE™ Manuscripts

I, the Submitting Author has the right to grant and does grant on behalf of all authors of the Work (as defined in the below author licence), an exclusive licence and/or a non-exclusive licence for contributions from authors who are: i) UK Crown employees; ii) where BMJ has agreed a CC-BY licence shall apply, and/or iii) in accordance with the terms applicable for US Federal Government officers or employees acting as part of their official duties; on a worldwide, perpetual, irrevocable, royalty-free basis to BMJ Publishing Group Ltd ("BMJ") its licensees and where the relevant Journal is co-owned by BMJ to the co-owners of the Journal, to publish the Work in BMJ Open and any other BMJ products and to exploit all rights, as set out in our licence.

The Submitting Author accepts and understands that any supply made under these terms is made by BMJ to the Submitting Author unless you are acting as an employee on behalf of your employer or a postgraduate student of an affiliated institution which is paying any applicable article publishing charge ("APC") for Open Access articles. Where the Submitting Author wishes to make the Work available on an Open Access basis (and intends to pay the relevant APC), the terms of reuse of such Open Access shall be governed by a Creative Commons licence – details of these licences and which Creative Commons licence will apply to this Work are set out in our licence referred to above.

Other than as permitted in any relevant BMJ Author's Self Archiving Policies, I confirm this Work has not been accepted for publication elsewhere, is not being considered for publication elsewhere and does not duplicate material already published. I confirm all authors consent to publication of this Work and authorise the granting of this licence.

For peer review only

Exclusion of enrolled participants in randomised controlled trials: what to do with ineligible participants?

Andrea M Rehman[1] Rashida A Ferrand[2,3] Elizabeth Allen[4] Victoria Simms[1,2] Grace McHugh[2] Helen A Weiss[1]

1. MRC Tropical Epidemiology Group, London School of Hygiene and Tropical Medicine, UK

2. Biomedical Research and Training Institute, 10 Seagrave Road, Harare, Zimbabwe

3. Department of Clinical Research, London School of Hygiene and Tropical Medicine, UK

4. Department of Medical Statistics, London School of Hygiene and Tropical Medicine, UK

Corresponding author: Andrea M Rehman

andrea.rehman@lshtm.ac.uk

For peer review only

For peer review only - http://bmjopen.bmj.com/site/about/guidelines.xhtml

Abstract

Objective

Post-randomisation exclusions in randomised controlled trials are common and challenge intention-to-treat principles. We summarise considerations for including or excluding data from participants identified as not meeting trial eligibility criteria after randomisation. We report how a decision might be reached and reported.

Key points

A key aim of randomisation is to minimize confounding by obtaining balance by both known and unknown potential confounding factors between trial arms. Including all enrolled participants in the primary analysis ensures an unbiased estimate of the intervention effect. However, ineligible participants are sometimes enrolled through errors in applying eligibility criteria, due to measurement or human error. Assuming there is no safety risk to ineligible participants enrolled, their inclusion in a trial and subsequent analysis of the intervention effect mirrors clinical practice. However, a randomised trial studies a specific population reflected by the eligibility criteria, and by including only eligible participants, clear recommendations can be made regarding intervention efficacy in this specific population. Consideration of the type of exclusion criteria not met in a trial will play a key role in deciding whether to include ineligible participants. Some criteria may be clearer cut, for example having the disease under study in a in a drug treatment trial. Other criteria may be less clear-cut. For example, in an treatment trial targeted at malnourished individuals, defined using an anthropometric cut-off, there may be measurement error, and some ineligible participants may be erroneously included.

Conclusion

The decision, by senior investigators, on whether to exclude enrolled participants, should reflect issues of safety, treatment efficacy, statistical power and measurement error. As long as decisions are made prior to finalising the statistical analysis plan for the trial, the risk of exclusions creating bias should be minimal. The decision taken should be transparently reported and a sensitivity analysis can present the opposite decision.

For peer review only

Background

The intention-to-treat (ITT) principle underpins the analysis of randomised controlled trials (RCTs) as a means of obtaining balance between arms on potential confounding factors, and preventing estimates of the intervention effect from being biased.[1, 2] ITT in its purest form estimate the intervention effect for all enrolled randomised participants, based on the initial arm allocation. In practice, the trial outcomes may not be measured or analysed for all randomised participants and this may impact on the ITT principle. One reason for not measuring outcomes in all enrolled participants is attrition, causing missing outcome data. If outcome data are missing not at random (MNAR) the ITT principle can become compromised.[3] A second reason is that some randomised participants may be excluded from analysis post-randomisation. This occurs commonly,[4] and again comprises the ITT principle. Reasons for such exclusions might be that participants (1) have incomplete baseline or outcome data, (2) did not receive the intervention allocated or (3) were found to be ineligible post-randomisation. In this communication, we discuss issues to consider when deciding whether to exclude enrolled, but ineligible, participants during the analysis of the intervention effect (Table 1).

Considerations

There is conflicting evidence as to whether post-randomisation exclusions of enrolled participants produce bias.[5-8] Bias can be considered a potential issue where decisions about exclusions are influenced by the trial sponsor or conflicts of interest of the investigators.[9]

The statistical power of the trial may be affected in either direction when ineligible individuals are enrolled incorrectly. Including the ineligible enrolled participants in analysis will retain a larger sample size, while excluding them may increase the variance of the estimated intervention effects (if those ineligible, were to respond differently to treatment than eligible participants).

The type of inclusion/exclusion criteria must be considered. For example, in a drug treatment trial providing treatment for a certain infection, if it was found post-randomisation that an enrolled participant was uninfected it is best to exclude that participant from analysis and withdraw them immediately from the trial. Decisions are less clear if i) the criteria include a cut-off used for inclusion (e.g. body mass index (BMI)), and there is error in the measurement of this, or ii) exclusion criteria include the presence of a clinical condition for which screening tests were not available at enrolment and only become apparent during follow-up.

Depending on how quickly it became apparent that a participant did not meet eligibility criteria, trial outcome data may have been collected on ineligible enrolled participants and a decision must be made whether to include them in primary analysis. If excluded, a “modified-ITT” may be performed.[10-12] We detail below the decision making during the bronchopulmonary function in response to azithromycin treatment for chronic lung disease in HIV-infected children (BREATHE) trial.[13]

Example

In an individually-randomised placebo-controlled trial of the impact of azithromycin on treatment of chronic lung disease in children and adolescents born with HIV in Zimbabwe and Malawi, one eligibility criteria was a measurement cut-off for lung function (using Forced Expiratory Volume in 1 second (FEV_1).[13] After enrolment was complete, and prior to data analysis, inconsistencies were identified with the FEV_1 inclusion. Height, one of the input variables for the reference equations on the European Respiratory Society/Global lung function initiative (ERS/GLI) 2012 spreadsheet,[14] as

For peer review only - http://bmjopen.bmj.com/site/about/guidelines.xhtml

measured at screening did not always align with height measurements from later study visits. At one study centre, on review of practices undertaken, it was noted that different models of stadiometers were used at different screening centres resulting in inconsistencies in height measurements. It was decided to recalculate the z-score using a mean of height from two later study visits in that country, and in the other country to use a mean height from screening and two later study visits (up to two weeks after randomisation). These recalculations meant that 11/347 (3.2%) participants fell outside the lung function cut-off for inclusion into the trial and a debate ensued among the trial investigators as to how to proceed. The first stage was to unmask these participants to the local study physician, report this protocol violation to the data safety and monitoring board (DSMB), and the trial steering committee (TSC) with a view to informing the relevant ethics committee(s).

At this stage, seven of the 11 ineligible participants had completed their course of study medication and the remaining four participants had between one and three weeks of study medication remaining. The trial drug, azithromycin, is considered safe,[15] so the potential for harm in continuing the four participants on treatment was considered low. The initial suggestion from the investigators was to withdraw these participants from the trial and stop treatment, but the DSMB and TSC advised that they should be included as the primary analysis, with a sensitivity analysis excluding them. The reason for this was i) lung function may vary by time of day,[16] so if lung function had been retested at a different time even on the same day, eligibility may have been different, ii) the low risk of treatment-related adverse events and iii) the advantage in retaining a greater sample size (original power calculations required 300 for primary outcome at 12 months). Further, a pre-specified subgroup analysis was included in the statistical analysis plan to investigate effect modification by baseline FEV_1 measurement, and provide estimates of treatment effect at different severities of baseline lung function. We also considered the possible adverse impact on statistical power if ineligible participants were less responsive to azithromycin treatment than eligible participants (potentially resulting in greater variability of treatment effect). On balance, this was outweighed by reasons to include ineligible participants.

Table 1: Reasons for and against post-randomisation exclusions

Issue	Reason to include	Reason to exclude
Maintain ITT principles, providing an unbiased treatment effect	Stays true to ITT principle ensuring balance on known and unknown factors between arms when all enrolled and randomised participants are analysed.	The risk of bias from excluding some participants has been shown to be low under certain conditions.
Make recommendations of benefit or harm (based on trial results) relating to a certain patient population	Where there is uncertainty over defining patient populations, it would be a conservative approach to retain all participants	Retains a defined group of included participants meeting inclusion/exclusion criteria neatly in which the intervention is hypothesised to be the most effective
The inclusion criteria are subject to measurement error. The relationship between the inclusion criteria and the primary outcome should be considered.	Pragmatically, errors in measurement will occur in routine practice. They may have been considered eligible at the point of enrolment. Consider whether measurement error in the inclusion criteria does not impact	Identification of errors in the measurement of disease state and excluding them can prevent underestimation of treatment effects.

For peer review only - http://bmjopen.bmj.com/site/about/guidelines.xhtml

	on the measurement of the primary outcome.	
Disease status may be unclear.	Measurement cut-off may not relate to a "disease" state and may be arbitrary.	Measurement cut-offs are commonly used to indicate disease severity albeit knowing there may be some misclassification.
Effect on statistical power	A larger sample size is retained.	If ineligible participants' responses to treatment differ from those for eligible participants (e.g. less response) the variance of the primary outcome may be increased meaning there may be more statistical power to exclude them.
Justifying the decision to include or exclude	Post-randomisation exclusions may be mistrusted in the scientific community if conflicts of interest or the trial sponsor are shown to have influenced the decision-making.	Post-randomisation exclusions are a common approach in the scientific community and will be accepted when clearly justified.
Assessment of safety risks	There is no safety risk to participants after review and therefore treatment and follow-up can continue.	Randomisation was mistakenly done, for example when found not to be diseased. Where safety was compromised the participants should cease remaining treatment and most likely be excluded from analysis.

Conclusion

There is not a one-size-fits-all approach to deciding on post-randomisation exclusions and in fact, there is evidence to suggest that more trials tend to make post-randomisation exclusions than do not.[4] Consideration should be given to safety, assessment of treatment effects, statistical power and measurement error (Table 1). We recommend that the decision is made after a joint discussion among senior trial investigators in conjunction with the trial steering committee and data and safety monitoring board. Others may advise, but the final decision falls to the senior investigators of the trial who should not be influenced by the trial sponsor or conflicts of interest, financial or otherwise. To further reduce bias a decision should be made *prior* to finalising the statistical analysis plan for the trial, and for transparency, reported explicitly when publishing the trial results. Justification for including or excluding the participants who were found not to meet inclusion criteria after randomisation should be presented for scrutiny by the scientific community and it may be appropriate to consider a sensitivity analysis using the opposite decision. The aim of any decision is to remain as close to ITT principles as possible and present an unbiased estimate of the treatment effect.

References

For peer review only

For peer review only - http://bmjopen.bmj.com/site/about/guidelines.xhtml

1. Fergusson, D., et al., *Post-randomisation exclusions: the intention to treat principle and excluding patients from analysis.* BMJ, 2002. **325**(7365): p. 652-4.
2. Soares, I. and A.V. Carneiro, *Intention-to-treat analysis in clinical trials: principles and practical importance.* Rev Port Cardiol, 2002. **21**(10): p. 1191-8.
3. Dumville, J.C., D.J. Torgerson, and C.E. Hewitt, *Reporting attrition in randomised controlled trials.* BMJ, 2006. **332**(7547): p. 969-71.
4. Abraha, I., et al., *A systematic review found that deviations from intention-to-treat are common in randomized trials and systematic reviews.* J Clin Epidemiol, 2017. **84**: p. 37-46.
5. Nuesch, E., et al., *The effects of excluding patients from the analysis in randomised controlled trials: meta-epidemiological study.* BMJ, 2009. **339**: p. b3244.
6. Schulz, K.F., et al., *Empirical evidence of bias. Dimensions of methodological quality associated with estimates of treatment effects in controlled trials.* JAMA, 1995. **273**(5): p. 408-12.
7. Schulz, K.F. and D.A. Grimes, *Sample size slippages in randomised trials: exclusions and the lost and wayward.* Lancet, 2002. **359**(9308): p. 781-5.
8. Yelland, L.N., et al., *Applying the intention-to-treat principle in practice: Guidance on handling randomisation errors.* Clin Trials, 2015. **12**(4): p. 418-23.
9. Montedori, A., et al., *Modified versus standard intention-to-treat reporting: are there differences in methodological quality, sponsorship, and findings in randomized trials? A cross-sectional study.* Trials, 2011. **12**: p. 58.
10. Abraha, I., et al., *Deviation from intention to treat analysis in randomised trials and treatment effect estimates: meta-epidemiological study.* BMJ, 2015. **350**: p. h2445.
11. Abraha, I. and A. Montedori, *Modified intention to treat reporting in randomised controlled trials: systematic review.* BMJ, 2010. **340**: p. c2697.
12. Kruse, R.L., et al., *Intention-to-treat analysis: who is in? Who is out?* J Fam Pract, 2002. **51**(11): p. 969-71.
13. Gonzalez-Martinez, C., et al., *Azithromycin versus placebo for the treatment of HIV-associated chronic lung disease in children and adolescents (BREATHE trial): study protocol for a randomised controlled trial.* Trials, 2017. **18**(1): p. 622.
14. Quanjer, P.H., et al., *Multi-ethnic reference values for spirometry for the 3-95-yr age range: the global lung function 2012 equations.* Eur Respir J, 2012. **40**(6): p. 1324-43.
15. Abu Dabrh, A.M., et al., *Prevention of exacerbations in patients with stable non-cystic fibrosis bronchiectasis: a systematic review and meta-analysis of pharmacological and non-pharmacological therapies.* BMJ Evid Based Med, 2018. **23**(3): p. 96-103.
16. Quanjer, P.H., et al., *GLI Advantages and Consequences*. 2012, Global Lung Function Initiative. p. 15.

Author Contributions

AMR prepared the first draft of manuscript. All authors were involved in the preparation and conception of the manuscript and critically reviewed the manuscript. All authors approved the submitted version.

Funding statement

The BREATHE trial was funded by the Global Health and Vaccination Research (GLOBVAC) Programme of the Medical Research Council of Norway. RAF is funded by the Wellcome Trust (Grant Number 206316/Z/17/Z). HAW, AMR and VS were supported in part by a grants from the Medical Research Council (MRC) and the Department for International Development (DFID UK) under the

For peer review only - http://bmjopen.bmj.com/site/about/guidelines.xhtml

MRC/DFID Concordat (K012126/1) and is also part of the EDCTP2 programme supported by the European Union.

Competing interests statement

The authors declare no competing interests.

For peer review only

For peer review only - http://bmjopen.bmj.com/site/about/guidelines.xhtml

Exclusion of enrolled participants in randomised controlled trials: what to do with ineligible participants?

Journal:	*BMJ Open*
Manuscript ID	bmjopen-2020-039546.R2
Article Type:	Communication
Date Submitted by the Author:	28-Oct-2020
Complete List of Authors:	Rehman, Andrea; London School of Hygiene and Tropical Medicine, MRC Tropical Epidemiology Group<br>Ferrand, Rashida; London School of Hygiene & Tropical Medicine, Department of Clinical Research; Biomedical Research and Training Institute<br>Allen, Elizabeth; London School of Hygiene & Tropical Medicine, Department of Medical Statistics<br>Simms, Victoria; London School of Hygiene and Tropical Medicine, MRC Tropical Epidemiology Group; Biomedical Research and Training Institute<br>McHugh, Grace; Biomedical Research and Training Institute, Harare<br>Weiss, Helen; London School of Hygiene and Tropical Medicine, MRC Tropical Epidemiology Group
**Primary Subject Heading**:	Research methods
Secondary Subject Heading:	Epidemiology
Keywords:	STATISTICS & RESEARCH METHODS, Paediatric thoracic medicine < THORACIC MEDICINE, EDUCATION & TRAINING (see Medical Education & Training)

SCHOLARONE™
Manuscripts

I, the Submitting Author has the right to grant and does grant on behalf of all authors of the Work (as defined in the below author licence), an exclusive licence and/or a non-exclusive licence for contributions from authors who are: i) UK Crown employees; ii) where BMJ has agreed a CC-BY licence shall apply, and/or iii) in accordance with the terms applicable for US Federal Government officers or employees acting as part of their official duties; on a worldwide, perpetual, irrevocable, royalty-free basis to BMJ Publishing Group Ltd ("BMJ") its licensees and where the relevant Journal is co-owned by BMJ to the co-owners of the Journal, to publish the Work in BMJ Open and any other BMJ products and to exploit all rights, as set out in our licence.

The Submitting Author accepts and understands that any supply made under these terms is made by BMJ to the Submitting Author unless you are acting as an employee on behalf of your employer or a postgraduate student of an affiliated institution which is paying any applicable article publishing charge ("APC") for Open Access articles. Where the Submitting Author wishes to make the Work available on an Open Access basis (and intends to pay the relevant APC), the terms of reuse of such Open Access shall be governed by a Creative Commons licence – details of these licences and which Creative Commons licence will apply to this Work are set out in our licence referred to above.

Other than as permitted in any relevant BMJ Author's Self Archiving Policies, I confirm this Work has not been accepted for publication elsewhere, is not being considered for publication elsewhere and does not duplicate material already published. I confirm all authors consent to publication of this Work and authorise the granting of this licence.

For peer review only

Exclusion of enrolled participants in randomised controlled trials: what to do with ineligible participants?

Andrea M Rehman[1] Rashida A Ferrand[2,3] Elizabeth Allen[4] Victoria Simms[1,2] Grace McHugh[3] Helen A Weiss[1]

1. MRC International Statistics and Epidemiology Group, London School of Hygiene and Tropical Medicine, UK

2. Department of Clinical Research, London School of Hygiene and Tropical Medicine, UK

3. Biomedical Research and Training Institute, 10 Seagrave Road, Harare, Zimbabwe

4. Department of Medical Statistics, London School of Hygiene and Tropical Medicine, UK

Corresponding author: Andrea M Rehman

andrea.rehman@lshtm.ac.uk

For peer review only

Abstract

Objective

Post-randomisation exclusions in randomised controlled trials are common and may include participants identified as not meeting trial eligibility criteria after randomisation. We report how a decision might be reached and reported on, to include or exclude these participants. We illustrate using a motivating scenario from the BREATHE trial (Trial registration ClinicalTrials.gov, NCT02426112) evaluating azithromycin for the treatment of chronic lung disease in 6-19 year olds with HIV in Zimbabwe and Malawi.

Key points

Including all enrolled and randomised participants in the primary analysis of a trial ensures an unbiased estimate of the intervention effect using intention to treat principles, and minimises the effects of confounding through balanced allocation to trial arm. Ineligible participants are sometimes enrolled, due to measurement or human error. Of 347 participants enrolled into the BREATHE trial, 11 (3.2%) were subsequently found to be ineligible based on lung function criteria. We assumed no safety risk of azithromycin treatment; their inclusion in the trial and subsequent analysis of the intervention effect therefore mirrors clinical practice. Senior trial investigators considered diurnal variations in the measurement of lung function, advantages of retaining a higher sample size and advice from the data safety and monitoring board and trial steering committee, and decided to include these participants in primary analysis. We planned and reported analyses including and excluding these participants, and in our case the interpretation of treatment effect was consistent.

Conclusion

The decision, by senior investigators, on whether to exclude enrolled participants, should reflect issues of safety, treatment efficacy, statistical power and measurement error. As long as decisions are made prior to finalising the statistical analysis plan for the trial, the risk of exclusions creating bias should be minimal. The decision taken should be transparently reported and a sensitivity analysis can present the opposite decision.

For peer review only

Motivating example

In an individually-randomised placebo-controlled trial (registered ClinicalTrials.gov, NCT02426112) of the impact of azithromycin on treatment of chronic lung disease in children and adolescents born with HIV in Zimbabwe and Malawi, one eligibility criteria was a measurement cut-off for lung function (using Forced Expiratory Volume in 1 second (FEV_1) z-score).[1, 2] After enrolment was complete, and prior to data analysis, inconsistencies were identified with the FEV_1 inclusion criteria. Specifically, height (an input variable for the reference equations on the European Respiratory Society/Global lung function initiative (ERS/GLI) 2012 spreadsheet to compute the FEV1 z-score,[3]) measured at screening did not always align with height measurements from later study visits. A review of practices undertaken found that in one country, different models of stadiometers were used at different screening centres resulting in inconsistencies in height measurements. It was decided to recalculate the z-score using a mean of height from two later study visits in that country, and in the other country to use a mean height from screening and two later study visits (up to two weeks after randomisation). These recalculations meant that 11/347 (3.2%) participants fell outside the lung function cut-off for inclusion into the trial and a debate ensued among the trial investigators as to how to proceed. The first stage was to unmask these participants to the local study physician, report this protocol violation to the data safety and monitoring board (DSMB), and the trial steering committee (TSC) and to the relevant ethics committee(s).

At this stage, seven of the 11 ineligible participants had completed their course of study medication and the remaining four participants had between one and three weeks of study medication remaining. The trial drug, azithromycin, is considered safe,[4] so the potential for harm in continuing the four participants on treatment was considered low. The initial suggestion from the investigators was to withdraw these participants from the trial and stop treatment, but the DSMB and TSC advised that they should be included as the primary analysis, with a sensitivity analysis excluding them. The reason for this was i) lung function may vary by time of day,[5] so if lung function had been retested at a different time even on the same day, eligibility may have been different, ii) the low risk of treatment-related adverse events and iii) the advantage in retaining a greater sample size (original power calculations required 300 for primary outcome at 12 months). Further, a pre-specified subgroup analysis was included in the statistical analysis plan to investigate effect modification by baseline FEV_1 measurement, and provide estimates of treatment effect at different severities of baseline lung function. We also considered the possible adverse impact on statistical power if ineligible participants were less responsive to azithromycin treatment than eligible participants (potentially resulting in greater variability of treatment effect). On balance, this was outweighed by reasons to include ineligible participants.

Statistical considerations

The intention-to-treat (ITT) principle underpins the analysis of randomised controlled trials (RCTs) as a means of obtaining balance between arms on potential confounding factors, and preventing estimates of the intervention effect from being biased.[6, 7] ITT in its purest form estimate the intervention effect for all enrolled randomised participants, based on the initial arm allocation. In practice, the trial outcomes may not be measured or analysed for all randomised participants and this may impact on the ITT principle. One reason for not measuring outcomes in all enrolled participants is attrition, causing missing outcome data. If outcome data are missing not at random (MNAR) the ITT principle can become compromised.[8] A second reason, which compromises the ITT principle, but is common nonetheless,[9] is that some randomised participants may be excluded from analysis post-randomisation. Reasons for such exclusions might be that participants (1) have incomplete baseline or outcome data, (2) did not receive the intervention allocated or (3) were

For peer review only - http://bmjopen.bmj.com/site/about/guidelines.xhtml

For peer review only

87 found to be ineligible post-randomisation. In this communication, we summarise issues to consider
88 when deciding whether to exclude enrolled, but ineligible, participants during the analysis of the
89 intervention effect (Table 1).

90 Table 1: Reasons for and against post-randomisation exclusions

**Issue**	**Reason to include**	**Reason to exclude**
Clinical scenario		
Make recommendations of benefit or harm (based on trial results) relating to a certain patient population	Where there is uncertainty over defining patient populations, it would be a conservative approach to retain all participants	Retains a defined group of included participants meeting inclusion/exclusion criteria neatly in which the intervention is hypothesised to be the most effective
Disease status may be unclear.	Measurement cut-off may not relate to a “disease” state and may be arbitrary.	Measurement cut-offs are commonly used to indicate disease severity albeit knowing there may be some misclassification.
Assessment of safety risks	There is no safety risk to participants after review and therefore treatment and follow-up can continue.	Randomisation was mistakenly done, for example when found not to be diseased. Where safety was compromised the participants should cease remaining treatment and most likely be excluded from analysis.
Statistical analysis		
Maintain ITT principles, providing an unbiased treatment effect	Stays true to ITT principle ensuring balance on known and unknown factors between arms when all enrolled and randomised participants are analysed.	The risk of bias from excluding some participants has been shown to be low under certain conditions.
The inclusion criteria are subject to measurement error. The relationship between the inclusion criteria and the primary outcome should be considered.	Pragmatically, errors in measurement will occur in routine practice. They may have been considered eligible at the point of enrolment. Include if measurement of the primary outcome is not impacted by measurement error in the inclusion criteria.	Identification of errors in the measurement of disease state and excluding them can prevent underestimation of treatment effects.
Effect on statistical power	A larger sample size is retained.	If ineligible participants’ responses to treatment differ from those for eligible participants (e.g. less response) the variance of the primary outcome may be increased meaning there may be more statistical power to exclude them.
Integrity & transparency		
Justifying the decision to include or exclude	Post-randomisation exclusions may be mistrusted in the scientific	Post-randomisation exclusions are a common approach in the

For peer review only

	community if conflicts of interest or the trial sponsor are shown to have influenced the decision-making.	scientific community and will be accepted when clearly justified.

There is conflicting evidence as to whether post-randomisation exclusions of enrolled participants produce bias.[10-13] Bias can be considered a potential issue where decisions about exclusions are influenced by the trial sponsor or conflicts of interest of the investigators.[14]

The statistical power of the trial may be affected in either direction when ineligible individuals are enrolled incorrectly. Including the ineligible enrolled participants in analysis will retain a larger sample size, while excluding them may increase the variance of the estimated intervention effects (if those ineligible, were to respond differently to treatment than eligible participants).

The type of inclusion/exclusion criteria must be considered. For example, in a drug treatment trial providing treatment for a certain infection, if it was found post-randomisation that an enrolled participant was uninfected it is best to exclude that participant from analysis and withdraw them immediately from the trial. Decisions are less clear if i) the criteria include a cut-off used for inclusion (e.g. body mass index (BMI)), and there is error in the measurement of this, or ii) exclusion criteria include the presence of a clinical condition for which screening tests were not available at enrolment and only become apparent during follow-up.

Depending how quickly it became apparent that a participant did not meet eligibility criteria, trial outcome data may have been collected on ineligible enrolled participants and a decision must be made whether to include them in primary analysis. If excluded, a “modified-ITT” may be performed.[15-17]

Reporting and reflection on motivating example

The primary outcome was analysed for 308 participants, 11 of whom were ineligible based on FEV_1 inclusion criteria. By chance, differences were observed between trial arms in age and sex distributions and with HIV-related characteristics. Primary analyses were therefore pre-specified, prior to unmasking of outcome data, to adjust for site, age, sex, and HIV viral load. Once-weekly administration of azithromycin did not improve lung function measured by FEV_1 z-score after 48 weeks in intention to treat analysis (adjusted mean difference (aMD) 0.06 – 95% Confidence interval -0.10 to 0.21) and in sensitivity analysis excluding those who did not meet eligibility criteria (aMD 0.07 – 95% CI -0.08 to 0.23).[2] The pre-specified per protocol analysis suggested weak evidence for an effect of azithromycin, with an aMD in z-scores of 0.14 (95% CI -0.02 to 0.29) favouring azithromycin. Those not meeting eligibility criteria were more likely to be in the azithromycin arm, in Malawi, younger, of male sex and have HIV viral suppression (Table 2).

Table 2: Baseline characteristics of BREATHE trial participants stratified by eligibility for inclusion

Characteristic	Eligible randomised participants n=336	Eligible analysed participants n=297	Ineligible randomised and analysed participants n=11[1]
Placebo arm, n (%)	170 (51)	142 (48)	4 (36)
AZM arm, n (%)	166 (49)	155 (52)	7 (64)

For peer review only - http://bmjopen.bmj.com/site/about/guidelines.xhtml

Baseline FEV_1 z-score, mean (SD)	-2.05 (0.72)	-2.05 (0.73)	-0.67 (0.38)
48-week FEV_1 z-score, mean (SD)	-	-1.95 (0.90)	-1.24 (0.84)
Zimbabwe site, n (%)	241 (72)	219 (74)	0 (0)
Malawi site, n (%)	95 (28)	78 (26)	11 (100)
Aged 6-10, n (%)	44 (13)	40 (13)	3 (27)
Aged 11-15, n (%)	152 (45)	135 (45)	6 (55)
Aged 16-19, n (%)	140 (42)	122 (41)	2 (18)
Female sex, n (%)	166 (49)	142 (48)	4 (36)
Male sex, n (%)	170 (51)	155 (52)	7 (64)
Baseline log10 HIV viral load, mean (SD)[2]	2.79 (1.61)	2.72 (1.59)	2.32 (1.95)
Baseline suppressed HIV viral load (<1000 copies/ml), n (%)[2]	187 (56)	171 (58)	7 (64)

[1] All ineligible randomised participants were analysed for the primary outcome

[2] n=2 missing values among eligible participants were imputed in the primary analysis using chained equations

The study was powered to detect a 0.32 z-score difference between trial arms with 300 participants assuming a mean z-score of -2.04 (standard deviation 0.82) in the placebo arm. The primary outcome was assessed in 308 participants, with a mean of -1.95 (SD 0.91) in the placebo arm. Effectively, with a sample size of 146 in the placebo arm and 162 in the azithromycin arm the study had 80% power to detect a 0.29 z-score difference between trial arms; excluding ineligible participants gave the same z-score difference.

In practice, the inclusion of ineligible participants did not change the interpretation of the trial results, likely due to their low numbers and/or because the adjustments used for primary analysis (to account for baseline imbalance) were also associated with ineligibility (and being assessed for the primary outcome). The study remained sufficiently powered. Sensitivity analyses were pre-specified in a formal statistical analysis plan, shared with reviewers and reported in the publication of the trial findings for transparency and to maintain research integrity.

Conclusion

There is not a one-size-fits-all approach to deciding on post-randomisation exclusions and in fact, there is evidence to suggest that more trials tend to make post-randomisation exclusions than do not.[9] Consideration should be given to safety, assessment of treatment effects, statistical power and measurement error (Table 1). We recommend that the decision is made after a joint discussion among senior trial investigators in conjunction with the trial steering committee and data and safety monitoring board. Others may advise, but the final decision falls to the senior investigators of the trial who should not be influenced by the trial sponsor or conflicts of interest, financial or otherwise. To further reduce bias a decision should be made *prior* to finalising the statistical analysis plan for the trial, and for transparency, reported explicitly when publishing the trial results. Justification for including or excluding the participants who were found not to meet inclusion criteria after randomisation should be presented for scrutiny by the scientific community and it may be appropriate to consider a sensitivity analysis using the opposite decision. The aim of any decision is to remain as close to ITT principles as possible and present an unbiased estimate of the treatment effect.

For peer review only - http://bmjopen.bmj.com/site/about/guidelines.xhtml

References

1. Gonzalez-Martinez, C., et al., *Azithromycin versus placebo for the treatment of HIV-associated chronic lung disease in children and adolescents (BREATHE trial): study protocol for a randomised controlled trial.* Trials, 2017. **18**(1): p. 622.
2. Ferrand, R.A., et al., *Once-weekly azithromycin versus placebo in children with HIV-associated chronic lung disease (BREATHE): a randomized, clinical trial.* JAMA Network Open, 2020. **Accepted**.
3. Quanjer, P.H., et al., *Multi-ethnic reference values for spirometry for the 3-95-yr age range: the global lung function 2012 equations.* Eur Respir J, 2012. **40**(6): p. 1324-43.
4. Abu Dabrh, A.M., et al., *Prevention of exacerbations in patients with stable non-cystic fibrosis bronchiectasis: a systematic review and meta-analysis of pharmacological and non-pharmacological therapies.* BMJ Evid Based Med, 2018. **23**(3): p. 96-103.
5. Quanjer, P.H., et al., *GLI Advantages and Consequences*. 2012, Global Lung Function Initiative. p. 15.
6. Fergusson, D., et al., *Post-randomisation exclusions: the intention to treat principle and excluding patients from analysis.* BMJ, 2002. **325**(7365): p. 652-4.
7. Soares, I. and A.V. Carneiro, *Intention-to-treat analysis in clinical trials: principles and practical importance.* Rev Port Cardiol, 2002. **21**(10): p. 1191-8.
8. Dumville, J.C., D.J. Torgerson, and C.E. Hewitt, *Reporting attrition in randomised controlled trials.* BMJ, 2006. **332**(7547): p. 969-71.
9. Abraha, I., et al., *A systematic review found that deviations from intention-to-treat are common in randomized trials and systematic reviews.* J Clin Epidemiol, 2017. **84**: p. 37-46.
10. Nuesch, E., et al., *The effects of excluding patients from the analysis in randomised controlled trials: meta-epidemiological study.* BMJ, 2009. **339**: p. b3244.
11. Schulz, K.F., et al., *Empirical evidence of bias. Dimensions of methodological quality associated with estimates of treatment effects in controlled trials.* JAMA, 1995. **273**(5): p. 408-12.
12. Schulz, K.F. and D.A. Grimes, *Sample size slippages in randomised trials: exclusions and the lost and wayward.* Lancet, 2002. **359**(9308): p. 781-5.
13. Yelland, L.N., et al., *Applying the intention-to-treat principle in practice: Guidance on handling randomisation errors.* Clin Trials, 2015. **12**(4): p. 418-23.
14. Montedori, A., et al., *Modified versus standard intention-to-treat reporting: are there differences in methodological quality, sponsorship, and findings in randomized trials? A cross-sectional study.* Trials, 2011. **12**: p. 58.
15. Abraha, I., et al., *Deviation from intention to treat analysis in randomised trials and treatment effect estimates: meta-epidemiological study.* BMJ, 2015. **350**: p. h2445.
16. Abraha, I. and A. Montedori, *Modified intention to treat reporting in randomised controlled trials: systematic review.* BMJ, 2010. **340**: p. c2697.
17. Kruse, R.L., et al., *Intention-to-treat analysis: who is in? Who is out?* J Fam Pract, 2002. **51**(11): p. 969-71.

Author Contributions

AMR prepared the first draft of manuscript. AMR, RAF, EA, VS, GM, and HAW were involved in the preparation and conception of the manuscript, critically reviewed the manuscript and approved the submitted version.

Funding statement

For peer review only - http://bmjopen.bmj.com/site/about/guidelines.xhtml

The BREATHE trial was funded by the Global Health and Vaccination Research (GLOBVAC) Programme of the Medical Research Council of Norway. RAF is funded by the Wellcome Trust (Grant Number 206316/Z/17/Z). HAW, AMR and VS were supported in part by a grants from the Medical Research Council (MRC) and the Department for International Development (DFID UK) under the MRC/DFID Concordat (K012126/1) and is also part of the EDCTP2 programme supported by the European Union.

Competing interests statement

The authors declare no competing interests.

For peer review only

For peer review only - http://bmjopen.bmj.com/site/about/guidelines.xhtml
